# Topological Alterations of Working Memory Impairment in Aged Patients With Vascular Dementia

**DOI:** 10.3389/fnagi.2021.741445

**Published:** 2021-10-04

**Authors:** Cao Zheng, Rong-Sheng Zhang, Ting Wan, Jun-Sheng Zhao

**Affiliations:** ^1^Department of Radiation Intervention, Central Hospital of Huanggang City, Huanggang, China; ^2^Department of Radiology, Central Hospital of Huanggang City, Huanggang, China

**Keywords:** aSAH, vascular dementia, working memory deficit, independent component analysis, small-world

## Abstract

Aneurysmal subarachnoid hemorrhage (aSAH) is a common disease causing vascular dementia. Survivors often suffer from cognitive impairment especially working memory deficit. Currently, lack of theoretical support limits the improvement of cognitive intervention or rehabilitation. It is unclear how the large-scale network differs and to what extent is the brain network affected? Our study aims to provide novel information about the topological characteristics of brain organization, especially “small-world” property. A total of 62 aSAH patients are enrolled in this study. They are divided into two groups according to the syndrome of working memory deficit. Their working memory function is evaluated by TMT-B and AVLT (Chinese version). Functional MRI scan is also performed for detecting resting-state cortical plasticity. We utilized ICA to extract functional sub-networks including working memory network from imaging data. And then we establish binarized network and calculate the small-worldness property as well as local and global efficiency of networks. aSAH group with working memory deficit shows no significant difference of clustering coefficient with control group. Our study discovered significant decrease of characteristic path length indicating an increase of overall routing efficiency. We reason that patients with working memory deficit have to recruit more neuronal resources and thus develops higher overall routing efficiency of local network. This study provides novel information about the neural alterations of aSAH patients with working memory deficit. It might contribute to the understanding of neural mechanism and the improvement of current intervention for vascular dementia.

## Introduction

Aneurysmal subarachnoid hemorrhage (aSAH) is one common disease which causes vascular dementia. It is traditionally regarded as one severe incident with high mortality and morbidity (Lovelock et al., 2010). The hemorrhagic strike is sudden and deadly because of a high prevalence of occult or undetected intracranial aneurysms (Vernooij, 2007; Vlak et al., 2011). But in the recent decades, there is a tremendous improvement on the overall survival rate of aSAH patients due to the great progress made on early diagnosis and timely intervention.

As the mortality rate is dropping in the last two decades, more patients experience severe neurological sequela after survival. Several studies reported that even those survivors categorized into “mild disability” also suffered from various cognitive impairments such as working memory deficit, aphasia or alexia (Ørbo et al., 2008). That increases the number of patients who needs rehabilitative care and return to their normal life. As a result, it is of great value to study the cognitive impairment after aSAH. New strategy or improvement of intervention and rehabilitation needs theoretic foundation, which comes from specific description of neural behavior and recovery process.

According to the previous research, short-term memory has been listed as the most important sequela (Mayer et al., 2002; Anderson et al., 2006; Al-Khindi et al., 2010; Scott et al., 2010). Particularly, reports pointed out that working memory deficit is often affected (Sheldon et al., 2012, 2013). This persistent cognitive problem lays a great impact on patients' daily life. Although a number of rehabilitative methods have been applied, most approaches are empirical or experimental (Wong et al., 2016; Huenges Wajer et al., 2017; Stabel et al., 2017). Lacking theoretical support somehow limits the improvement of current rehabilitation. Of course, previous studies have provided insight into the functional alterations of the working memory deficit after aSAH. It reported that aSAH patients with cognitive impairment displayed significant decline of spontaneous neural activity in several cerebral regions (Su, 2018). However, there are still questions about the specific cortical changes of aSAH patients with working memory deficit. (1)To what extent is the brain network of those with memory deficit affected, whole-brain network level or sub-network level? (2) How is the working memory network organized when isolated regions have been proved dysfunctional? Although the existing evidence revealed some functional changes, identifying worthwhile large-scale changes would be a vital advance for clarifying the underlying mechanism of working memory deficit.

In this study, we would extract several sub-networks including working memory network from the resting-state fMRI data. The topological changes of networks instead of activation status of one brain region would be analyzed. We aim to provide novel information about the characteristics of brain organization, especially “small-world” property (Sporns and Zwi, 2004). The comparison would be performed in the aSAH patients with and without working memory deficit in order to reveal more information about the associated neural plasticity.

## Materials and Methods

### Demographics and Ethic Information

A total of 62 aSAH patients who are treated and followed up in our center are identified for neuropsychological assessment and MR scan, including 32 with intact working memory and another 30 with working memory deficit. We only recruit those patients with over 1 year follow-up so that their outcome of cognitive impairment would be identified. Loss Trail Making Test part B (TMT-B) and AVLT (the Chinese version of AVLT, Auditory Verbal Learning Test) is performed for evaluation of cognitive function. Participants' original demographic and clinical status at first admission are retrospectively adopted and compared. The severity of aSAH is examined through head CT of Fisher grade (assessed by a neurologist and a radiologist). Definitive treatment of clipping or coiling is performed within 48 h after first admission.

We select MRI-safe titanium clips as implants in order to produce minimal artifact (Khursheed, 2011). An advanced 3T MR scanner can successfully image brain tissue around implanted titanium aneurysm clips at different spatial ranges depending on the sequence type. The patients enrolled in this study had lesions distant from the regions of interest on fMRI analysis. Most of the clips had limited artifacts 2 mm outside of the boarder. In addition, construction of a spatial confidence boundary of signal integrity, represented numerically by an artifact mask volume, was performed. It was possible to quantify the degree of uncertainty across patients regarding whether the signal in a particular region of the brain was completely or partially affected by clip artifacts.

Inclusion criteria of this study is listed as follows: (1) aged over 18 years; (2) aneurysm diagnosed through digital subtraction angiography; (3) minimum 1 year of follow-up since admission; (4) Right handedness, evaluated by Edinburgh laterality manual test. The exclusion criteria includes: (1) History of aSAH related complications (hydrocephalus, rebleeding or vasospasm); (2) other cerebral surgical or interventional treatment before enrollment; (3) Disturbances in the motor, sensitive or cerebellum assessment. This study is approved by the ethics committee of Ruijin Hospital, Shanghai Jiaotong University Medical School. All participants provided written informed consent according to the Helsinki Statement.

Patients who survived the hemorrhagic attack spent circa 20 days in the neurology and neurosurgery department and another 60 days in a rehabilitation center. Neuroimaging and other tests were performed after discharge and initial rehabilitation, which was usually 75–90 days after admission to our center. A washout period of at least 30 days was used to eliminate the effect of previous medication.

### Data Acquisition and Preprocessing

We conduct this study with a GE 3.0T MR System. The parameters of scans are listed as followed: sequence = GRE-EPI, axial slices, scanning order = interleaved [1:2:43 2:2:42], slice number = 43, matrix size = 64 ^*^ 64, FOV = 192 ^*^ 192 mm, TR/TE = 2,000/30 ms, FA = 90 deg, slice thickness = 3.0 mm, gap = 0 (voxel size 3.0 ^*^ 3.0 ^*^ 3.0), dummy scan = 6, number of acquisitions = 240, NEX = 1, parallel acceleration = 2, total scan time = 8 min 12 s.

### Data Preprocessing

We utilize Statistical Parametric Mapping toolbox (https://www.fil.ion.ucl.ac.uk/spm/) for data preprocessing. The functional data is first adjusted with slicing time procedure for difference of acquisition time. Then head movement correction procedure is performed. Those subjects of which the translational or rotational motion over 2.5 mm or 2.5 degrees would be discarded. In the procedure of realignment, one mean volume is extracted and pointed as the reference image. We perform spatial normalization of the functional images via standard EPI template. Covariates including head motion parameters, white matter and cerebral spinal fluid BOLD signal would be regressed from functional data. Finally, spatial smoothing with a Gaussian kernel of 6-mm full width at half maximum is performed for denoising.

### Independent Component Analysis

We utilize GICA toolbox (Group ICA of fMRI Toolbox, Generally, group independent component analysis with concatenation approach plus back-reconstruction is utilized for these multi-subject analysis (Calhoun et al., 2001). We first perform dimension reduction to the functional data. Afterwards, the number of independent components of subjects decreases to 60. Then temporal concatenation is performed for image connection. All the images are reduced to 40 components at group level with EM (expectation maximization) algorithm. Also, we perform 100 times repetition of infomax algorithm in ICASSO for better robustness. After aggregated spatial maps are estimated, the back reconstruction approach is utilized for extracting subject-specific spatial pattern and time courses. We threshold these maps of spatial weight with a significance level of *p* < 0.05. After revealing spatial pattern of certain component, we manually recognize eight sub-networks including working memory network and so on. This component which shows a spatial pattern of working memory network would be binarized and transformed into a mask. It helps to extract time series of working memory network from the original data. In this procedure, we also determine the spatial coordinate of several peak points which represents the highest possibility of belonging to the current component.

### Node and Edge Definition

We aim to explore the difference of small-world property between two groups at both whole brain network and sub-network level. For whole brain network analysis, we use random parcellation and segment the brain into 1,000 nodes. It was proved that the topological property did not depend on the methodology of parcellation (Zalesky et al., 2010). Then, we calculate the functional connectivity between each two nodes. The Pearson correlation of mean time courses in each two nodes are considered as edge.

For sub-network analysis, we first perform an independent component analysis and extract 40 spatial components for the construction of sub-network. Then we manually recognize eight sets of independent components representing eight sub-networks, including basal ganglia network (BGN), dorsal default mode network (d-DMN), visual network (VN), left executive control network (LECN), sensorimotor network (SMN), visual spatial network (VSN), anterior-salience network (a-SN) and working memory network (WMN). For each sub-network, we define each voxel as node and voxel-voxel functional connectivity as edge. In order to balance the scale of edge number of two levels, we adjust the voxel size and resample it into 6 mm^3^, which massively reduces the computational work. The networks constructed in this study are all undirected and unweighted. In the calculation, we set the sparsity from 0.05 to 0.50 with an increased step of 0.01.

### Analysis of Small-Worldness in Working Memory Network

The major metrics of small-world network involved in this study are clustering coefficient (Cp), characteristic path length (Lp), normalized clustering coefficient γ and normalized characteristic path length λ. Cp of network is calculated as the mean of the clustering coefficients of all the nodes in the network. Clustering coefficient quantifies the extent of local interconnectivity or cliquishness of a network. Decreased Cp implies reduced efficiency in local information transmission and processes (Wang et al., 2016). The mean minimum path length of a node is computed as the average of minimum distances from that node to all the remaining nodes in the network. Lp of network is the average of the mean minimum path lengths of all the nodes in the network. Characteristic path length Lp measures the extent of overall communication efficiency of a network and increased Lp represents a shift toward “regularization” (Suo et al., 2017). As for the isolated nodes in the network, which are totally disconnected with the network, is assigned 0 for clustering coefficient and Inf for path length. For normalization purpose, we also construct random networks as baseline. We calculate the ration of Cp and Lp of target network and same metrics of random network with the same number of nodes and degree distribution (Sporns and Zwi, 2004). In contrast to random network, a network with small-world property has a high normalized clustering coefficients γ(Cp/Cprand) > 1 and relatively low normalized characteristic path length λ(Lp/Lprand) ≈ 1 (Achard, 2006).

### Analysis of Global and Local Efficiency of Whole Brain Network and Sub-networks

The global efficiency of network is the inverse of the harmonic mean of the minimum path length between each pair of nodes. According to the previous studies, global efficiency is a more meaningful measure in parallel information processing than path length (Achard and Bullmore, 2007). On the other hand, the local efficiency reflects the fault tolerance of a network by measuring the capability of its sub-network for information exchange when the index node are eliminated (Latora and Marchiori, 2001). Small-worldness properties, especially the path length is not robust if the network contains disconnected nodes. Therefore, we would also calculate the local and global efficiency of whole brain network as well as those sub-networks to provide supplementary information. Similarly, we create 100 random networks with same nodes number and degree as the target network. Then we compare the efficiency of target network with that of random network.

### Statistical Analysis

For metrics of graph theory, multiple comparison methods were applied by utilizing AUC (area under curve) as measurement. For numerical analysis, two sample *T* test was adopted. The statistical threshold was set at *p* < 0.05.

## Results

We compare the gender as well as the length of hospitalization and the statistical result shows no significant difference. The AVLT scaling result is 9.33 ± 0.93/12.45 ± 1.02(Mean ± SEM) in control group and 6.21 ± 0.76/9.58 ± 0.87 (Mean ± SEM) in working memory deficit group. The statistical analysis confirms significant decrease of delayed memory function in working memory deficit group (Table 1).

**Table 1:** Demographic information of enrolled aSAH subjects.

	**Patients without working memory deficit**	**Patients with working memory deficit**	**Statistical significance**
Age, mean (SEM^*^)	63.3 (3.3)	62.4 (4.6)	*P* > 0.05
Sex, male (%)	37.5% (*n* = 12)	43% (*n* = 13)	/
Length of hospitalization (days), mean (SEM)	21 (1.23)	23 (1.12)	*P* > 0.05
TMT-B (Second)	165 (45)	221 (72)	*P* < 0.05
AVLT (Chinese Version)-delayed memory	9.33 (0.9)	4.12 (1.1)	*P* < 0.05
AVLT (Chinese Version)-repeat recognition	12.45 (1.0)	7.26 (1.4)	*P* < 0.05
Total number of subjects	N = 32	N = 30	/

* *SEM, standard error of mean; TMT-B, trail making test part B; AVLT, auditory verbal learning test*.

**Figure 1:**
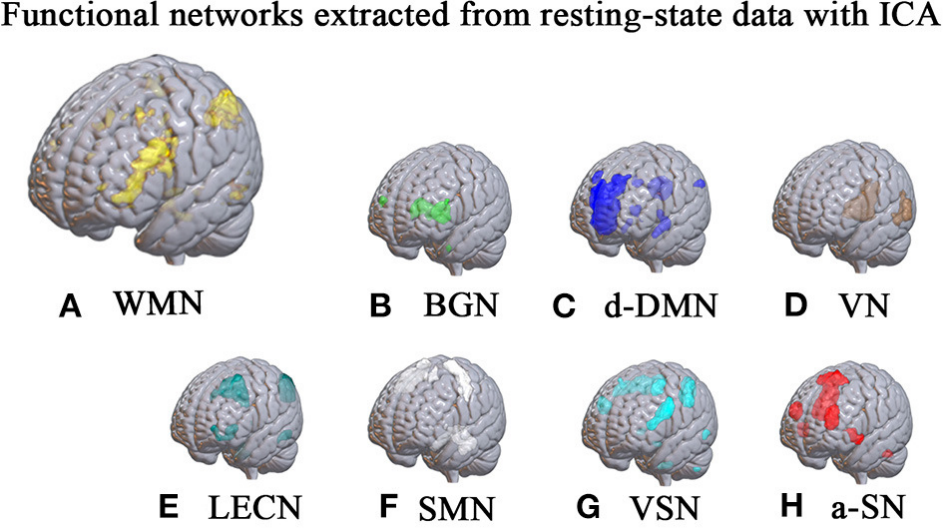
Functional networks extracted from resting-state data with ICA. With ICA approach, we manage to extract 40 components and establish totally 8 sub-networks from the resting data including working memory network **(A)**, basal ganglia network **(B)**, dorsal default mode network **(C)**, visual network **(D)**, left executive control network **(E)**, sensorimotor network **(F)**, visual spatial network **(G)** and anterior salience network **(H)**.

**Figure 2:**
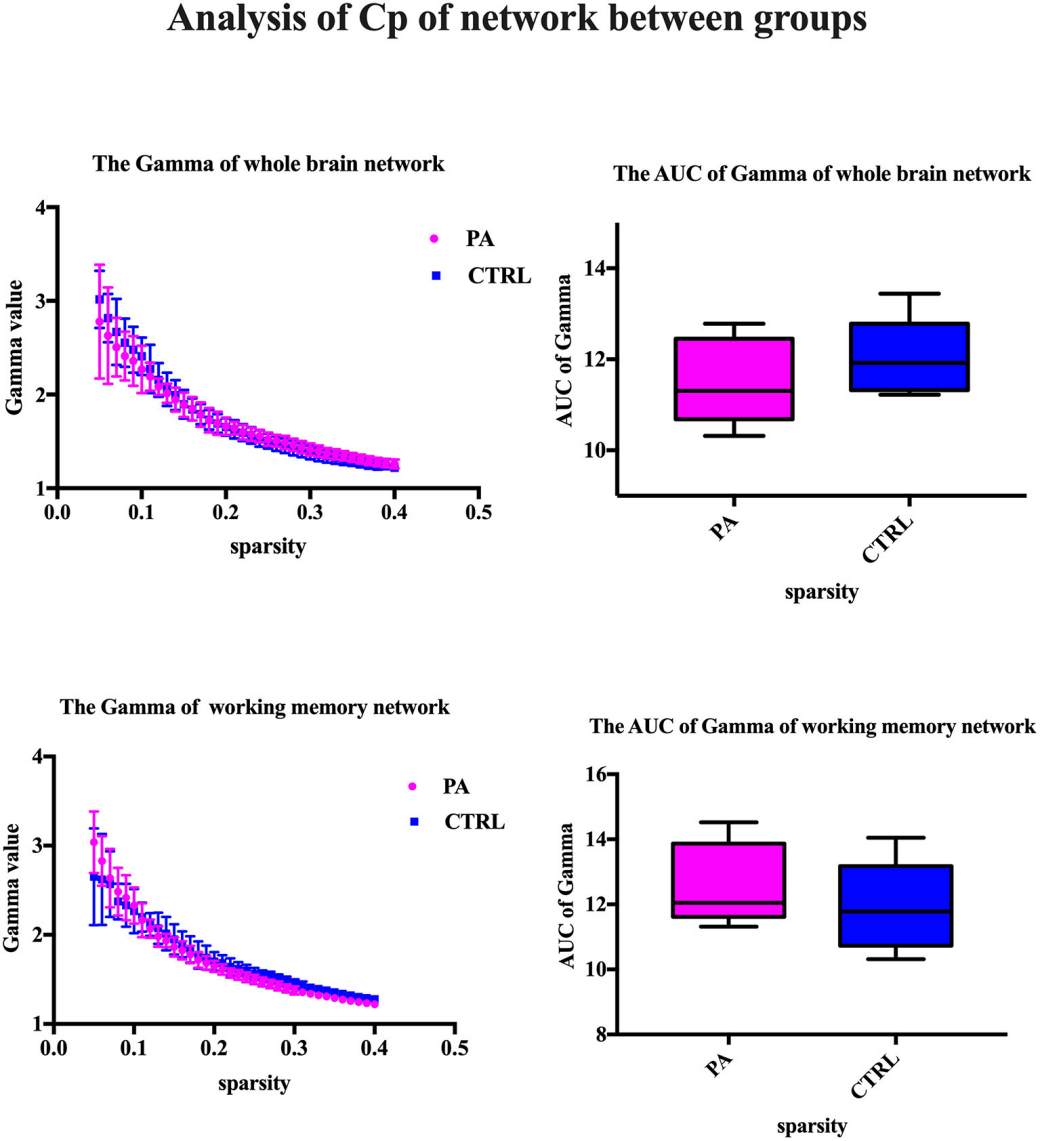
Analysis of Cp of network between groups. We compare the gamma value and its area under curve (AUC), which represents the clustering coefficient of whole brain network or working memory network. The statistical results show no significant difference.

**Figure 3:**
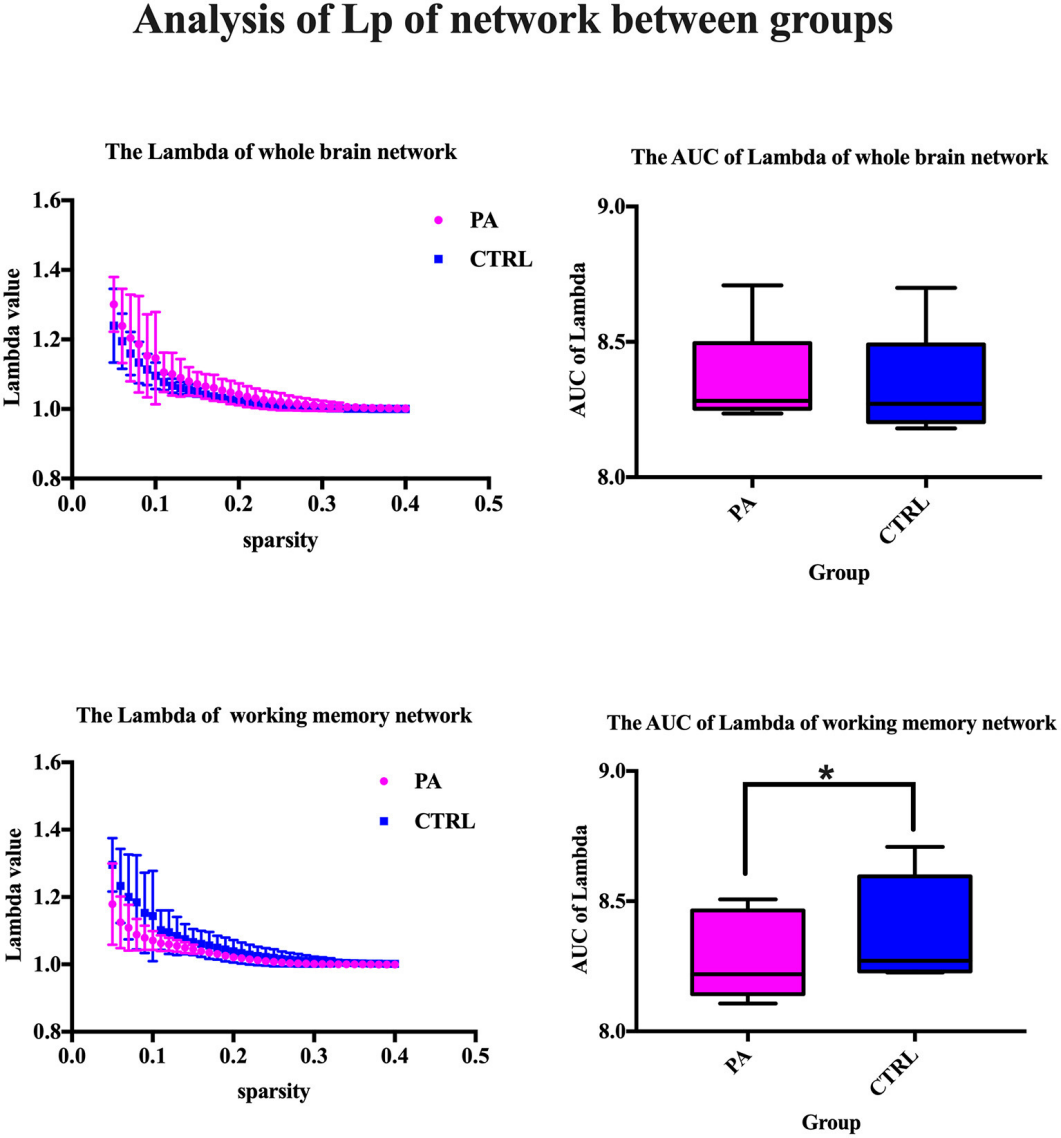
Analysis of Lp of network between groups. We compare the lambda value and its area under curve (AUC), which represents the characteristic path length of whole brain network or working memory network. The statistical results show significant decrease of characteristic path length in control group. *Significant difference of AUC of Lambda between two groups.

**Figure 4:**
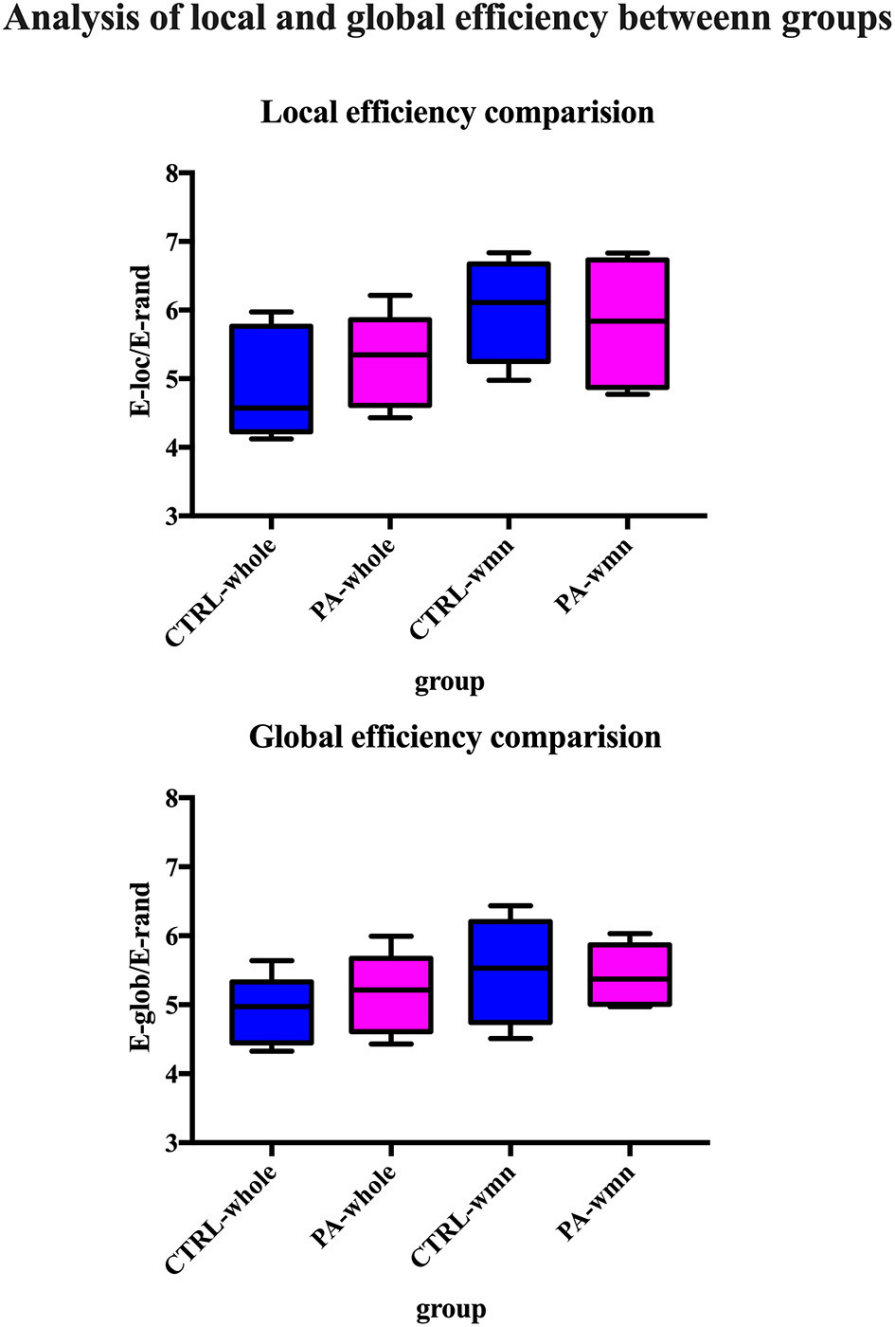
Analysis of local and global efficiency between groups. We compare both local and global efficiency of whole brain network of control group, whole brain network of PA group, working memory network of control group and working memory network of PA group. The statistical results show no significant difference among groups.

After ICA procedure, we extract 40 components and establish totally eight sub-networks from the resting data including working memory network, basal ganglia network, dorsal default mode network, visual network, left executive control network, sensorimotor network, visual spatial network (Figure 1). In the analysis of small-worldness analysis, aSAH group with working memory deficit shows no significant difference of clustering coefficient with control group (Figure 2). But it shows significant decrease of characteristic path length than control group (Figure 3). As for the analysis of local and global efficiency, the result shows no significant difference between groups (Figure 4).

## Discussion

The detailed alteration of cognitive impairment after aSAH has been always a vital problem for researchers (Welmer et al., 2006; Al-Khindi et al., 2010; Ali et al., 2020). Because improvement on current treatment or development of new interventional strategy depends on our understanding of neural mechanism. However, there is still a long way before the thorough clarification of neural basis, which leads to multiple cognitive impairment including working memory deficit. In fact, the exact neurological change of working memory deficit after aSAH remains unclear.

Our study is motivated by previous research, which indicated persistent memory dysfunction after recovery of aSAH (Ørbo et al., 2008; Schweizer and Macdonald, 2010). Task-dependent BOLD experiment has been utilized to reveal the abnormal cortical activation which is potentially associated with memory deficit (Ellmore et al., 2013). Nevertheless, it is still not sufficient for a detailed description on the neurological change. Because isolated activation of certain brain regions fails to give enough attention to the connective situation between those regions. It is possible to reveal more details about working memory deficit if we consider more about the characteristic of information transmission from an angle of brain network.

On the other hand, the cognitive impairment is believed to trigger very limited response in whole brain scale compared with other task of strong activation. In order to increase our sensitivity of detection, we also include regional functional network to our analysis. The working memory network is localized and identified by ICA, which enables us to extract individual sub-network from group-level data.

Our study provides insight to the topological property of working memory deficit after aSAH. The most important findings could be concluded as followed, (1) The significant alteration of working memory deficit stays in a limited extent, which lies in regional working memory network rather than whole-brain network. (2) Working memory dysfunction promotes higher overall routing efficiency of regional functional network.

The most important result of this study is the significant decrease of characteristic path length. That indicates the surprising increase of overall routing efficiency of working memory network in aSAH patients with working memory deficit, which somehow fails to be in line with former studies (Chung et al., 2019). Previous studies demonstrated that working memory deficit was related with reduced connectivity within the network (Arciniega et al., 2021; Vannest et al., 2021)and other neuron loss or dysfunctionality (Fang et al., 2018; Cheng et al., 2019). Most reports focus on the direct hemodynamic response or adjacent connectivity of regions such as left dorsolateral prefrontal cortex (DLPFC) (Burgess et al., 2010) or parietal cortex (Olesen et al., 2004). Some existing evidence suggested subjects with working memory deficit requires more executive control, and therefore more brain activity, compared to healthy (or faster) individuals in order to perform successfully (Postle et al., 1999; Chang et al., 2011). An exact idea to explain this is the Compensation-Related Utilization of Neural Circuits Hypothesis (Reuter-Lorenz and Cappell, 2008), whereby patients with working memory deficit have to recruit more neuronal resources at lower loads than those with normal memory capacity leaving fewer resources for processing at higher loads. Similar result was found in patients of ADHD with working memory deficit. The imaging study discovered decreased efficiency of DLPFC for high-load visuospatial working memory and greater reliance on posterior spatial attention circuits to store and update spatial position than healthy control youth (Bédard et al., 2014). Actually, it was unclear at the outset whether memory dysfunction would be accompanied by reduced or increased activation. But we could say that the direct task-dependent imaging and large-scale network analysis provide two dimension of information about the detailed neural alteration contributing to the working memory deficit. Of course, the current theory needs larger sample size for confirmation in further study.

We also noticed the insignificant working network in left hemisphere. Previous studies (Owen et al., 2005) reported activation in the left inferior parietal lobe and left precunues increased along with increasing age. Those regions were implicated in WMN studies. According to this theory, the subjects recruited in our study would display progressive recruitment of these task related frontal and parietal regions that underpin the functional maturation of working memory. In this circumstances, the left working memory system showed more robust architecture than the right one in the face of disease strike.

We perform independent component analysis for separating noise and functional sub-network. We concatenate all the data from both groups and then perform group-level dimensional reduction. It is true that the included images from two groups might differ in their brain signal. But we reason that the extracted spatial pattern of components should be unified in both groups for incoming statistical comparison. As a result, the spatial maps of component representing working memory network is not far different from previous human studies (Damoiseaux et al., 2006, 2008). In addition, our result does not include another pattern of working memory network, in which most of the significant voxels are localized in the left hemisphere. We think this may attribute to the limited sample size of included subjects. The component of right hemispheric working memory network fails to achieve the threshold of within-group significance.

## Conclusion

Working memory deficit is one severe symptom of vascular dementia patients such as aSAH survivors. Our study discovered significant decrease of characteristic path length, which indicates the increase of overall routing efficiency. We reason that patients with working memory deficit have to recruit more neuronal resources and thus develops higher overall routing efficiency of local network. This study provides novel information about the neural alterations of aSAH patients with working memory deficit. It might contribute to the understanding of its neural mechanism and the improvement of current intervention.

## Limitation

This study recruited limited number of subjects. However, the disease-triggered cognitive changes require larger sample size to determine.

## Data Availability Statement

The raw data supporting the conclusions of this article will be made available by the authors, without undue reservation.

## Ethics Statement

The studies involving human participants were reviewed and approved by IRB of Huanggang Central Hospital. The patients/participants provided their written informed consent to participate in this study. Written informed consent was obtained from the individual(s) for the publication of any potentially identifiable images or data included in this article.

## Author Contributions

CZ designed the study and wrote the article. R-SZ and TW analyzed the data. J-SZ reviewed this article. All authors contributed to the article and approved the submitted version.

## Conflict of Interest

The authors declare that the research was conducted in the absence of any commercial or financial relationships that could be construed as a potential conflict of interest.

## Publisher's Note

All claims expressed in this article are solely those of the authors and do not necessarily represent those of their affiliated organizations, or those of the publisher, the editors and the reviewers. Any product that may be evaluated in this article, or claim that may be made by its manufacturer, is not guaranteed or endorsed by the publisher.
